# Humanization in the management of hospitalized children and adolescents

**DOI:** 10.1186/1824-7288-40-S1-A31

**Published:** 2014-08-11

**Authors:** Gianluigi Gargantini

**Affiliations:** 1 Department of Pediatrics, Lodi, 26900, Italy

## 

The rights of infants and children in hospital, which are universally accepted from an ideological point of view, result as being only partially applied and in a non-homogenous way in the different realities[1-3]. Many countries have set standards which would allow the translation of these principles into clinical practice. In 2008 the Italian Pediatric Society (SIP) and the Foundation for Children in Hospital, with the support of Progea & Joint Commission International, drafted a specific manual in which the requirements to be satisfied are clearly expressed and in which they also started up a voluntary accreditation programme of paediatric departments. The humanization of hospitals is a continually evolving process in relation to the changing needs of children and to the context in general. The current reference model is the Patient and Family Centered Care (PFCC) [4]. Intervention is required at all levels.

Training: this represents the foundation of the process and should examine the numerous aspects such as communication, management of relationships, cultural competence and teamwork.

Organization: activation of alternative assistance models, or models integrated to hospital admission; admission into spaces which are separated from those of adults (it occurs in 72.2% of subjects between 0-17 years of age and in 16% of those between 15 and 17); dedicated paths and guaranteed paediatric treatment in all areas of assistance, including the emergency area; family centered round care which represents the hospital paradigm of the PFCC concept in which the multidisciplinary discussion is carried out within the room, in the presence of the child and the family, integrating their outlook in the decision process; organizational flexibility and in the procedures [5].

Structure: the wards should be designed with the concept of a “paediatric hospital” within a general hospital where minors are cared for in dedicated areas and with sufficient flexibility to respond to the various needs. The optimal number of single rooms is a subject of debate. For adolescents, separated areas which safeguard privacy and independence are essential. Spaces for support activities and family members’ comfort must be provided for (table 1).

**Table 1:** SIP SURVEY OF 237 PEDIATRIC WARDS – 2010

***ASPECTS OF HOSPITALITY***	***%***
NO SINGLE ROOM	23

ADOLESCENT HOSPITALIZATION AREA	26

GAMES ROOM	91

ADOLESCENT ENTERTAINMENT AREA	23

SCHOOL	53

VOLUNTEER ASSOCIATION	61

AVAILABILITY OF A CULTURAL MEDIATOR	74

TRANSLATION TELEPHONE SERVICE	19

Information and education: aimed at actively involving the children and the families in the decisions and in the treatment, but also at understanding one’s rights.

It is fundamental to have a strong local leadership that drives the change with group work, alliances with institutions and involving children and families. Validated measures of process and outcome are necessary.

## References

[B1] Foster M Whitehead L Maybee P Cullens V The parents', hospitalized child's and health care providers' perceptions and experiences of family centered care within a pediatric critical care setting. A metasynthesis of qualitative research J Fam Nurs 2013 19 431 468 10.1177/1074840713496317 23884697

[B2] Kelly M Jones S Wilson V Lewis P How children’s rights are constructed in family-centered care: a review of the literature J Child Health Care 2012 16 190 205 10.1177/1367493511426421 22363046

[B3] Migone M Nicholas F Lennon R Are we following the European charter? Children, parents and staff perceptions Child Care Health Dev 2008 34 409 417 10.1111/j.1365-2214.2008.00822.x 18462456

[B4] Committee on Hospital Care and Institute for Patient and Family Centered Care Patient-and family-centered care and the pediatrician’s role Pediatrics 2012 129 394 404 22291118 10.1542/peds.2011-3084

[B5] Mittal V Sigrest T Ottolini M Rauch D Lin H Kit B Landrigan c Flores G Family-centered rounds on pediatric wards: a PRIS network survey of US and Canadian hospitalists Pediatrics 2010 126 37 43 10.1542/peds.2009-2364 20587682

